# PADAPT 1.0 – the Pannonian Dataset of Plant Traits

**DOI:** 10.1038/s41597-023-02619-9

**Published:** 2023-10-25

**Authors:** Judit Sonkoly, Edina Tóth, Nóra Balogh, Lajos Balogh, Dénes Bartha, Kinga Csendesné Bata, Zoltán Bátori, Nóra Békefi, Zoltán Botta-Dukát, János Bölöni, Anikó Csecserits, János Csiky, Péter Csontos, István Dancza, Balázs Deák, Zoltán Konstantin Dobolyi, Anna E-Vojtkó, Ferenc Gyulai, Alida Anna Hábenczyus, Tamás Henn, Ferenc Horváth, Mária Höhn, Gusztáv Jakab, András Kelemen, Gergely Király, Szabolcs Kis, Gergely Kovacsics-Vári, András Kun, Éva Lehoczky, Attila Lengyel, Barbara Lhotsky, Viktor Löki, Balázs András Lukács, Gábor Matus, Andrea McIntosh-Buday, Attila Mesterházy, Tamás Miglécz, Attila Molnár V, Zsolt Molnár, Tamás Morschhauser, László Papp, Patrícia Pósa, Tamás Rédei, Dávid Schmidt, Ferenc Szmorad, Attila Takács, Júlia Tamás, Viktor Tiborcz, Csaba Tölgyesi, Katalin Tóth, Béla Tóthmérész, Orsolya Valkó, Viktor Virók, Tamás Wirth, Péter Török

**Affiliations:** 1 Department of Ecology, University of Debrecen, Debrecen, Hungary; 2 HUN-REN-UD Functional and Restoration Ecology Research Group, Debrecen, Hungary; 3 Natural History Department, Savaria Museum, Szombathely, Hungary; 4 Institute of Environmental Protection and Nature Conservation, University of Sopron, Sopron, Hungary; 5 Herman Ottó Institute Nonprofit Ltd, Budapest, Hungary; 6 Department of Ecology, University of Szeged, Szeged, Hungary; 7 1016 Budapest, Piroska u. 4., Budapest, Hungary; 8 Institute of Ecology and Botany, HUN-REN Centre for Ecological Research, Vácrátót, Hungary; 9 Department of Ecology, University of Pécs, H-7624 Pécs, Ifjúság u. 6., Pécs, Hungary; 10 Institute for Soil Sciences, HUN-REN Centre for Agricultural Research, Budapest, Hungary; 11 1039 Budapest, Gyula u. 9., Budapest, Hungary; 12 Lendület Seed Ecology Research Group, Institute of Ecology and Botany, HUN-REN Centre for Ecological Research, Vácrátót, Hungary; 13 Hungarian Natural History Museum, Budapest, Hungary; 14 Institute of Botany, Czech Academy of Sciences, Třeboň, Czech Republic; 15 Institute for Wildlife Management and Nature Conservation, Hungarian University of Agriculture and Life Sciences, Gödöllő, Hungary; 16 József Attila City Library and Museum Collection, Komló, Hungary; 17 Department of Botany, Hungarian University of Agriculture and Life Sciences, Budai Campus, Budapest, Hungary; 18 Department of Environmental and Landscape Geography, Eötvös Loránd University, Budapest, Hungary; 19 Institute of Silviculture and Forest Protection, University of Sopron, Sopron, Hungary; 20 Department of Botany, University of Debrecen, Debrecen, Hungary; 21 HUN-REN-UD Conservation Biology Research Group, Debrecen, Hungary; 22 1115 Budapest, Halmi u. 5. 3/16., Budapest, Hungary; 23 Department of Environmental Sustainability, Institute of Environmental Sciences, Hungarian University of Agriculture and Life Sciences, Georgikon Campus, H-8360 Keszthely, Deák F. u. 16, Keszthely, Hungary; 24 National Food Chain Safety Office, Budapest, Hungary; 25 Wetland Ecology Research Group, Institute of Aquatic Ecology, HUN-REN Centre for Ecological Research, Debrecen, Hungary; 26 ÖMKi - Hungarian Research Institute of Organic Agriculture, Budapest, Hungary; 27 Doctoral School of Biology, Faculty of Sciences, University of Pécs, Pécs, Hungary; 28 Botanical Garden, University of Debrecen, Debrecen, Hungary; 29 Balaton-felvidéki National Park Directorate, Csopak, Hungary; 30 Department of Plant Systematics, Ecology and Theoretical Biology, Eötvös Loránd University, Budapest, Hungary; 31 Department of Botany, Hungarian Natural History Museum, Budapest, Hungary; 32 Saint Orsolya Catholic Secondary and Elementary School, Sopron, Hungary; 33 MTA-SZTE ‘Momentum’ Applied Ecology Research Group, University of Szeged, Szeged, Hungary; 34 HUN-REN-UD Biodiversity and Ecosystem Services Research Group, Debrecen, Hungary; 35 Aggtelek National Park Directorate, Jósvafő, Hungary; 36 Botanical Garden, University of Pécs, Pécs, Hungary; 37 Polish Academy of Sciences, Botanical Garden - Center for Biological Diversity Conservation in Powsin, Warszawa, Poland

**Keywords:** Ecology, Community ecology

## Abstract

The existing plant trait databases’ applicability is limited for studies dealing with the flora and vegetation of the eastern and central part of Europe and for large-scale comparisons across regions, mostly because their geographical data coverage is limited and they incorporate records from several different sources, often from regions with markedly different climatic conditions. These problems motivated the compilation of a regional dataset for the flora of the Pannonian region (Eastern Central Europe). PADAPT, the Pannonian Dataset of Plant Traits relies on regional data sources and collates data on 54 traits and attributes of the plant species of the Pannonian region. The current version covers approximately 90% of the species of the region and consists of 126,337 records on 2745 taxa. By including species of the eastern part of Europe not covered by other databases, PADAPT can facilitate studying the flora and vegetation of the eastern part of the continent. Although data coverage is far from complete, PADAPT meets the longstanding need for a regional database of the Pannonian flora.

## Background & Summary

The trait-based approach has been significantly advancing our ecological and evolutionary understanding in various fields of research including vegetation science^1,2^. To support trait-based analyses with suitable data, several international plant trait databases have been established in the last decades. Some of them compile data for a wide range of traits at the regional scale, e.g., BiolFlor for the German flora^3^, LEDA for Northwest Europe^4^, BROT for the flora of the Mediterranean^5^, or AusTraits for the Australian flora^6^. Other databases provide data for a specific group of traits at the global scale, e.g., SID, the Seed Information Database^7^, D^3^, the Dispersal and Diaspore Database^8^, and SylvanSeeds, a germination database of deciduous forests^9^. There are also databases covering not only the traits and attributes of the flora of a region but also its vegetation, such as PLADIAS, the Database of the Czech Flora and Vegetation^10^. TRY^11^, the most frequently used (meta-)database, incorporates several databases, thus providing a global coverage for numerous plant traits.

Although data on a broad range of traits can be relatively easily retrieved from these databases, the fact that they incorporate records from several different sources, often from regions with markedly different climatic conditions, sometimes also with varying measurement standards is a shortcoming for certain analyses. Climate and local abiotic conditions can cause substantial intraspecific trait variability^12^, which renders the application of such broad-scale databases problematic for regional-scale studies. Moreover, the existing European databases’ geographical coverage is limited and mostly focused either on the flora of the western and north-western part of Europe, or on the southern, mostly Mediterranean parts of the continent^5,13^.

Our dataset aims to cover the flora of the Pannonian biogeographic region which is situated in the eastern part of Central Europe surrounded by the Carpathians, the Alps, and the Dinaric Mountains. For the purpose of the dataset, we considered the Pannonian biogeographic region in the broadest sense; thus, we considered all areas included in any one of the overlapping territories of the Pannonian vegetation region, the Pannonicum floristic region or the Pannonian biogeographic region recognised by the European Union (for an overview see Fekete *et al*.^14^). The whole territory of Hungary is included in this region, along with some rather small, typically lowland areas of Slovakia, the Czech Republic, Austria, Slovenia, Croatia, Serbia, Romania, and Ukraine. Our main reason for choosing the described region was that the territory of Hungary does not match with any (bio)geographical region, and it is more reasonable and meaningful for the dataset to cover a biogeographical region rather than a political entity such as a country.

Due to the above-mentioned geographical focus of the existing databases, a great proportion of the Pannonian flora is not represented in them. These problems limit the applicability of the existing databases for not just studies of the Pannonian flora and vegetation, but for studies in the eastern and central part of the continent in general, and also for studies attempting large-scale comparisons across regions.

The above-mentioned issues have motivated the compilation of a dataset focusing on the Pannonian flora. Here, we introduce PADAPT 1.0, the Pannonian Dataset of Plant Traits, which relies on regional data sources and collates data on a wide range of traits and attributes of the Pannonian flora and makes it broadly available to the international scientific community.

PADAPT 1.0 provides regionally collected data and covers a high number of species with continental, Balkanic, and Pontic distributions; thus, it will promote studies of the flora and vegetation of not only the Pannonian region, but also the whole eastern half of the continent. The new dataset highlights data gaps, facilitates their targeted filling and promotes the exploration of intraspecific trait variability. Data collection will continue in the future and the PADAPT team welcomes any researcher interested in contributing to PADAPT with new regional data. In the coming years we expect to release PADAPT 2.0 complemented with additional attributes and further species.

## Methods

The checklist of taxa was based on the checklist of the Distribution atlas of vascular plants of Hungary^15^ which is constantly being updated with the species newly discovered in the country and therefore it is the most up-to-date checklist of the flora of Hungary. We adopted the checklist of the distribution atlas as of 1st January 2020, and then we used this checklist to harmonise the data coming from different sources. The current version of the dataset, PADAPT 1.0 only includes species of the Pannonian region that occur within the territory of Hungary, but trait measurements were in part carried out on specimens collected outside of Hungary^16,17^. As several of the data sources do not distinguish between subspecies, subspecies are generally not distinguished in the dataset either, except in cases when just one subspecies occurs in Hungary (e.g., *Astragalus vesicarius* subsp. *albidus*).

Along with the initialisation of collecting existing data, the PADAPT team started a sampling campaign in the Pannonian region to allow for measurements of leaf traits and seed mass in order to expand the range of existing trait data. The PADAPT protocol for measuring seed mass and leaf traits was based on data standards for LEDA^4^ and the protocols by Perez-Harguindeguy *et al*.^18^. These trait data have already been published^16,17^ and resulted in new leaf trait records for 1156 species^16^ and new thousand-seed mass (TSM) data for 281 species^17^ that have been incorporated into PADAPT.

To incorporate in the dataset, we considered data published in books and peer reviewed articles. There are four exceptions: the IUCN Red List category of species, and Conservation status (in Hungary), Conservation value (HUF), and Year of protection (in Hungary) which are based on a ministerial decree currently in force in Hungary (see Table 1). To reduce the effect of intraspecific variability and different climatic conditions in other regions, we aimed to focus on trait data measured in the Pannonian region and did not consider publications which contain trait data for the species in the PADAPT checklist but from different regions. Some of the attributes are based on a single data source, while for some other attributes we collated data from several different published sources (see Tables 1, 2). The dataset includes multiple columns for traits for which we have data from multiple sources (thousand-seed mass, seed bank persistence, and all leaf traits). The published sources that were used to collect the data differ in their approach regarding the presentation of the measured values. Some of them only present the mean of multiple measurements (e.g., the mean of three leaves of each of ten individuals), while some of them contain more data points per species (e.g., presenting the means of leaves coming from different individuals separately). We decided to unify the approaches of the different sources by taking one mean value per data source per species. In most cases it results in one data point per species per locality. We applied a different approach only with chromosome numbers and ploidy levels because these data originate from 33 different published sources and most of these sources contain data for only a very limited set of the species.

**Table 1:** Overview of the 54 attributes included in PADAPT 1.0: Attributes related to growth habit, strategy, reproduction, dispersal, karyology, distribution, and conservation (continued in Table 2).

Attribute name	Type	Units	Data coverage	Relevant columns in the dataset^25^	Data sources
Minimum height	Cont.	cm	95.6%	1	^26^
Maximum height	Cont.	cm	95.9%	2	^26^
Raunkiær life form	Categ.	—	96.2%	3	^26^
Social Behaviour Type	Categ.	—	80.7%	4	^24^
Ujvárosi’s life form system	Categ.	—	28.8%	5	^27^
Modified Ellenberg—Müller-Dombois life form system	Categ.	—	89.6%	6–7	^28^
Onset of flowering	Ord.	Months	96.1%	8	^26^
Duration of flowering	Ord.	Months	96.1%	9	^26^
Thousand-seed mass (TSM)	Cont.	g	67.1%	10–14	^17,20,21,29–31^
Seed mass category	Ord.	—	80.7%	15	^19–21^
Seed bank persistence	Ord.	—	19.7%	16–23	^19,32–36^
Seed bank persistence index	Cont.	—	19.7%	24	^19,32–36^
Dispersal strategy	Categ.	—	100%	25	^22^
Chromosome number	List*	—	83.7%	26	^37–69^
Ploidy level	Categ.	—	3.1%	27	^37–69^
Phytosociological categorization (Soó)	Categ.	—	73.2%	28	^23,24,70–118^
Phytosociological categorization (Borhidi)	Categ.	—	75.0%	29	^23,24,70–118^
Nativeness	Categ.	—	100%	30	^119^
Residence time	Categ.	—	100%	31	^119^
Introduction mode	Categ.	—	100%	32	^119^
Invasion status	Categ.	—	100%	33	^119^
Area type	Categ.	—	82.2%	34	^70^
Conservation status (in Hungary)	Categ.	—	100%	35	Decree 13/2001 KÖM
Conservation value (HUF)	Categ.	HUF	100%	36	Decree 13/2001 KÖM
Year of protection (in Hungary)	Categ.	Year	100%	37	Decree 13/2001 KÖM
IUCN Red List category	Categ.	—	100%	38	https://www.iucnredlist.org/
Hungarian Red List category	Categ.	—	100%	39	^120^
Conservation value category	Categ.	—	99.7%	40	^121^

Notations: Categ. – categorical variable; Cont. – continuous variable; Ord. – Ordinal variable. *A list of chromosome numbers of the species reported in the literature.

**Table 2:** Overview of the 54 attributes included in PADAPT 1.0: Ecological indicator values and leaf traits (continued from Table 1).

Attribute name	Type	Units	Data coverage	Relevant columns in the dataset^25^	Data sources
Ellenberg’s L (EIC)	Ord.	—	64.5%	41	^122^
Ellenberg’s T (EIC)	Ord.	—	64.5%	42	^122^
Ellenberg’s K (EIC)	Ord.	—	64.5%	43	^122^
Ellenberg’s F (EIC)	Ord.	—	64.5%	44	^122^
Ellenberg’s R (EIC)	Ord.	—	64.5%	45	^122^
Ellenberg’s N (EIC)	Ord.	—	64.5%	46	^122^
Ellenberg’s S (EIC)	Ord.	—	64.5%	47	^122^
Zólyomi’s T (EIC)	Ord.	—	62.5%	48	^70,123^
Zólyomi’s W (EIC)	Ord.	—	63.4%	49	^70,123^
Zólyomi’s R (EIC)	Ord.	—	62.2%	50	^70,123^
Soó’s T (EIC)	Ord.	—	76.9%	51	^23,70^
Soó’s F (EIC)	Ord.	—	77.0%	52	^23,70^
Soó’s R (EIC)	Ord.	—	75.0%	53	^23,70^
Soó’s N (EIC)	Ord.	—	72.8%	54	^23,70^
Borhidi’s T (EIC)	Ord.	—	77.1%	55	^24^
Borhidi’s W (EIC)	Ord.	—	77.1%	56	^24^
Borhidi’s R (EIC)	Ord.	—	77.1%	57	^24^
Borhidi’s N (EIC)	Ord.	—	77.1%	58	^24^
Borhidi’s L (EIC)	Ord.	—	77.1%	59	^24^
Borhidi’s C (EIC)	Ord.	—	77.1%	60	^24^
Borhidi’s S (EIC)	Ord.	—	77.1%	61	^24^
Leaf area (LA)	Cont.	mm^2^	57.8%	62–65	^16,124–126^
Leaf dry matter content (LDMC)	Cont.	mg/g	57.4%	66–69	^16,124–126^
Specific leaf area (SLA)	Cont.	mm^2^/mg	57.4%	70–73	^16,124–126^
Leaf fresh mass	Cont.	g	55.6%	74–75	^16,125^
Leaf dry mass	Cont.	mg	55.4%	76–77	^16,125^

Notations: Categ. – categorical variable; Cont. – continuous variable; Ord. – Ordinal variable; EIC – Ecological indicator value.

In case of seed mass category, we were able to categorise further species into the system of Csontos^19^ based on recent publications containing data from seed mass measurements carried out in Hungary^20,21^. Seed bank persistence index was calculated as the ratio of data indicating a persistent soil seed bank with a value from 0 to 1, where zero means that all available published data indicates a transient seed bank, and 1 means that all data indicates a persistent seed bank. In case of dispersal strategy, species that have not been evaluated by Sádlo *et al*.^22^ were categorised into the same dispersal strategies according to the descriptions provided in Sádlo *et al*.^22^. Some of the species previously not categorised into Soó’s and Borhidi’s phytosociogical system^23,24^ were also assigned a phytosociological category based on literature data (see Table 1).

A trait or attribute was included in PADAPT if appropriate data was available for it for a considerable proportion of the checklist and if it was considered meaningful for future ecological studies. After all these considerations, we have compiled the dataset based on 109 published sources (Tables 1, 2).

## Data Records

The current version, PADAPT 1.0 consists of 126,337 individual records on 2745 taxa based on 109 different published sources. There are 54 attributes included in PADAPT. A summary of all the included traits and attributes is presented in Tables 1, 2. The dataset is available from figshare^25^ (10.6084/m9.figshare.21937157.v2) and the dataset file^25^ also includes a short explanation and data sources for every attribute.

## Technical Validation

Data included in PADAPT are based on published sources (peer-reviewed journal articles and books). Experts of the respective field were involved in the data compilation for every attribute to obtain greater accuracy and reliability. After the compilation of the dataset, all attributes were checked for errors by looking for abnormal values and outliers. In some cases, the issues were resolved by checking the original publications again or asking the author(s). Presumably erroneous values were omitted from the dataset. Despite the measures taken to attain high reliability, the occurrence of errors in the dataset is still possible and we encourage users to report any errors to the authors.

The PADAPT protocol for measuring seed mass and leaf traits was based on data standards for LEDA^4^ and the protocols by Perez-Harguindeguy *et al*.^18^ to ensure that the recently obtained trait data^16,17^ included in PADAPT are reliable and comparable to other databases.

The current version (1.0) includes only the species that can be found in the territory of Hungary. Although the number of species in the Pannonian flora is not established in the literature, we estimate that approximately 90% of the flora of the region is already represented in the current version.

## Usage Notes

Besides the dataset reposited in figshare^25^, data presented here is also available in an interactive form at the website www.padapt.eu.

All data included in PADAPT 1.0 are public, but the present paper should be appropriately referenced when using the data.

## Data Availability

No code was used to generate or process the data presented in this manuscript.
